# Treatment of *Haemophilus* bacteremia with benzylpenicillin is associated with increased (30-day) mortality

**DOI:** 10.1186/1471-2334-12-153

**Published:** 2012-07-09

**Authors:** Sara Thønnings, Christian Østergaard

**Affiliations:** 1 Department of Clinical Microbiology, Copenhagen University Hospital Herlev, Nordre Ringvej 75, Herlev, Denmark; 2 Department of Clinical Microbiology, Copenhagen University Hospital Hvidovre, Kettegård Alle 30, Hvidovre, DK-2650, Denmark

**Keywords:** *Haemophilus*, Bacteremia, Mortality, Antibiotic therapy, Acquisition, Charlson comorbidity index

## Abstract

**Background:**

Optimal antibiotic treatment strategies of *Haemophilus* infections are still needed. Therefore, 30-day case fatality rate (CFR) of Haemophilus bacteremia and efficacy of various antibiotic treatment regimes were studied.

**Methods:**

All episodes of Haemophilus bacteremia in the former Copenhagen County during the period 2000-9 were included in the study. Clinical and biochemical findings and outcome were collected retrospectively from medical records.

**Results:**

105 consecutive episodes were identified (median age: 69 years, with only 4 children <16 years), 72% were due to non-typeable -, 16% to typeable *H. influenzae*, and 11% to other *Haemophilus* species. Pneumonia was the most common primary focus (in 48%), and 58% of the patients had Charlson comorbidity index > 1. Definitive antibiotic therapy was in 26 cases benzylpenicillin, in 12 cases aminopenicillins, in 50 cases cefuroxime and in 16 cases broadspectrum antibiotics, whereas 1 palliative case died without start of therapy. Whereas the use of broadspectrum antibiotics was related to the severity of the disease (admittance to ICU, need for assisted ventilation or hemodialysis, septic shock), no significant difference in clinical features was demonstrated for therapy with benzylpenicillin, aminopenicillin or cefuroxime, except benzylpenicillin was rarely administered to immunosuppressed patients. The CFR was 22% (23/105). The choice of empiric antibiotic therapy was not significantly associated with mortality (adequate vs. inadequate treatment: 23% (21/93) vs. 17% (2/12), respectively, *P >* 0.05). In contrast, definite antibiotic therapy with cefuroxime or aminopenicillins resulted in a significantly lower CFR than treatment with benzylpenicillin (12% (6/50) or 0% (0/12) vs. 39% (10/26), respectively, Log rank test *P <* 0.02). When adjustments were made for other identified risk factors in bivariate logistic regression analysis, treatment with cefuroxime was still were found to be associated with a significantly lower CFR than for benzylpenicillin: OR: 0.21 (0.06-0.69), *P =* 0.01 (hospital-acquired bacteremia), OR: 0.27 (0.08-0.91), *P* = 0.04 (polymicrobial episodes), OR: 0.16 (0.04-0.59), *P =* 0.006 (admittance at intensive care unit), OR: 0.22 (0.06-0.82), *P =* 0.02 (alcohol abuse), OR: 0.15 (0.04-0.60), *P =* 0.008 (altered mental state), OR: 0.22 (0.07-0.71), *P =* 0.01 (temperature < 38 °C), OR: 0.23 (0.07-0.79), *P =* 0.02 (septic shock), OR: 0.21 (0.06-0.69), *P =* 0.01 (mechanical ventilation).

**Conclusion:**

Our results suggest that, after susceptibility testing, cefuroxime or aminopenicillins are preferable to benzylpenicillins as definitive therapy for *Haemophilus* bacteremia.

## Background

*Haemophilus* bacteremia still carries a high mortality, and clinical features such as gender, age, acquisition of the bacteremic episode, and the focus of infection have recently been demonstrated to be associated with an increased risk of a fatal outcome [1]. 3rd generation cephalosporins have been the recommended therapy for childhood meningitis due to *H. influenzae* type B, because of a more rapid sterilization of the cerebrospinal fluid than in therapy with aminopenicillins [2] or cefuroxime, and a better outcome of the disease with 3rd generation cephalosporins compared to cefuroxime [3]. Less is known, however, about the influence of various antibiotic treatment regimes on the outcome of other *Haemophilus* infections including bacteremia. In Denmark therapy with benzylpenicillin has been recommended for treatment of ampicillin-susceptible *Haemophilus* infections and has been considered to be as effective as other recommended treatment regimes (e.g. aminopenicillins and cefuroxime). These recommendations provided in some local Danish guidelines were to our knowledge primarily based on results obtained from time-kill experiments showing benzylpenicillin to be as active as amoxicillin against various *Haemophilus* species [4], whereas clinical studies comparing benzylpenicillin to other recommended treatment regimes for therapy of *Haemophilus* bacteremia still are lacking In addition, the European Committee on Antimicrobial Susceptibility Testing (EUCAST: http://www.eucast.org) has stated that there still are insufficient data for H. *influenzae* to set clinical breakpoints for benzylpenicillin [5]. Thus, optimal antibiotic treatment strategies for infections due to *Haemophilus* species are still needed.

This retrospective study aims to investigate the efficacy of benzylpenicillin therapy in comparison with cefuroxime/aminopenicillins treatment in 105 consecutive episodes of bacteremia due to *Haemophilus species* in the former Copenhagen County during the time period 2000–2009.

## Results

### Identification of Haemophilus bacteremia episodes

108 episodes of *Haemophilus* bacteremia were identified from 1 January 2000 to 31 December 2009. Three bacteremic episodes caused by *H. haemolyticus*, *H. parainfluenzae* and *H. influenzae* were considered contaminations, because the patients had no clinical evidence of bacteremia, and they survived without receiving any antibiotic therapy. Two patients had two episodes of *Haemophilus* bacteremia with different primary infection foci two and five years apart, respectively. These patients were included with both episodes. Thus the study compromised 105 episodes of *Haemophilus* bacteremia in 103 patients.

### Clinical features of Haemophilus bacteremia

Clinical and demographic characteristics according to *Haemophilus* grouping are shown as Additional file 1: Table S1 to the article. Almost all cases with *Haemophilus* bacteremia were adults (median age: 69 years (54–79)), with only 4 children under 16 years of age. A total of 58% of the bacteremic episodes were community-acquired, 30% were healthcare-related and 12% were hospital-acquired. Most bacteremic episodes were due to non-typeable *H. influenzae* (72%), whereas 16% were due to typeable *H. influenzae* (serotype-b (n = 11) or serotype-f (n = 6)) and 11% due to other *Haemophilus* species including *H. parainfluenzae* (n = 8), *H. aphrophilus* (n = 2), *H. haemolyticus* (n = 1) or *H. parahaemolyticus* (n = 1). There were 8 polymicrobial episodes, all due to non-typeable *H. influenzae*, with the isolation of respectively *Pseudomonas aeruginosa*, *Klebsiella pneumoniae*, *Staphylococcus aureus*/*Streptococcus pneumoniae*, *S. salivarius*, *S. anginosus* (x 2), *S. pneumoniae*, or other *Streptococcus species*. Most isolates were fully susceptible to ampicillin, cefuroxime and ciprofloxacin. However, 12 isolates were resistant to ampicillin, 2 to cefuroxime (minimal inhibitory concentration (MIC) = 4 and 8 mg/L), and 1 to cefotaxime (MIC = 0.25 mg/L).

A lung focus was the most common primary focus of infection (in 48%), and these episodes were predominantly due to non-typeable *H. influenzae* (94%). All 5 episodes with epiglottitis and 3 out of 4 meningitis episodes were caused by typeable *H. influenzae*, whereas all 3 episodes of endocarditis were due to *H. parainfluenzae*. The study population consisted of 44, 44 and 17 cases with a Charlson co-morbidity index of 0, 1–2 and 3, respectively. 25 patients had cancer (16 cases were haematological malignancies), 11 patients had moderate to severe renal disease, 12 had mild and 5 moderate to severe liver disease, 15 patients had chronic obstructive pulmonary disease, and 7 patients had diabetes. 22% (23/105) of the patients were immunosuppressed, and a significantly higher proportion of these patients had a healthcare-related bacteremia as compared to patients having a community-acquired bacteremia (45% (14/17) vs. 10% (6/61), respectively, *P <* 0.0001). 28% (21/76) of patients infected with non-typeable *H. influenzae* were immunosuppressed, which was a higher proportion than for typeable *H. influenzae* (6% (1/17)) and for other *Haemophilus species* (8% (1/12)). A significantly higher frequency of alcohol abuse was seen among patients with hospital-acquired bacteremia as compared to patients with community-acquired bacteremia (46% (6/13) vs. 11% (6/54), respectively, *P =* 0.008).

### Antibiotic treatment

Empiric antibiotic therapy at time of blood culturing was in 37 cases benzylpenicillin (15 of these in combination with gentamicin), in 7 cases aminopenicillins (3 of these in combination with gentamicin), in 41 cases cefuroxime (1 of these in combination with ampicillin, 8 with gentamicin and 1 with ciprofloxacin), in 5 cases piperacillin-tazobactam (3 of these in combination with gentamicin and 2 with ciprofloxacin), in 5 cases a 3rd generation cephalosporin (2 in combination with ampicillin), in 1 case meropenem, in 1 case ciprofloxacin, and in 1 case gentamicin alone. Dosage of antibiotics followed local guidelines (e.g. benzylpenicillin 1.2 g qid, ampicillin 1–2 g qid, cefuroxime 1.5 g tid for adults except for treatment of CNS infections and endocarditis that were treated with higher doses). 3 patients were initially treated with antibiotics not considered adequate (dicloxacillin, erythromycin), whereas 4 patients did not receive empiric antibiotic treatment. According to the susceptibility testing, empiric antibiotic therapy was not considered adequate in 2 additional episodes (1 treated with cefuroxime and 1 treated with penicillin), whereas 96 patients received adequate empiric antibiotic treatment).

Definitive antibiotic therapy chosen according to the susceptibility testing of the pathogen was in 26 cases benzylpenicillin (9 of these in combination with gentamicin), in 12 cases aminopenicillins (3 of these in combination with gentamicin), in 50 cases cefuroxime (10 of these in combination with gentamicin and 1 with ciprofloxacin), and in 15 cases broadspectrum antibiotics (piperacillin-tazobactam (n = 7; 4 of these in combination with gentamicin and 3 with ciprofloxacin), 3rd generation cephalosporin (n = 5), ciprofloxacin (n = 3), meropenem (n = 1)). 1 case with inoperable mouth cancer, who died 6 days after blood culturing, was palliative and did not receive any antibiotic therapy. The definitive antibiotic therapy was changed in 42 cases, whereas it was a continuation of the empiric antibiotic therapy in 62 cases, including the 5 cases where the patient died before telephone contact was made by a physician of the Department of Clinical Microbiology with the notification of the positive blood culture and the subsequent results of susceptibility testing. Among these 5 cases only one case, who was treated with benzylpenicillin, did not receive adequate definitive antibiotic therapy.

Clinical and demographic characteristics classified according to definitive antibiotic therapy are shown in Table 1. The use of broadspectrum antibiotics was related to the severity of the disease and was more frequently administered at the ICU (50% (8/16) vs. 12% (3/26) for benzylpenicillin (*P =* 0.01) or 16% (8/50) for cefuroxime (*P =* 0.02), respectively), to patients with septic shock (27% vs. 0% for cefuroxime, *P =* 0.002), to patients with meningitis (25% vs. 0% for benzylpenicillin and cefuroxime, *P =* 0.02), to patients given hemodialysis (19% vs. 2% for cefuroxime, P < 0.05) and to patients given assisted ventilation (38% vs. 8% for benzylpenicillin and cefuroxime, *P =* 0.04). In contrast, only 1 out of 23 immunosuppressed patients received treatment with benzylpenicillin (in combination with gentamicin), which was a significant lower proportion for benzylpenicillin as compared to treatment with cefuroxime (4% vs. 30%, respectively, *P* < 0.008) and with broadspectrum antibiotics (4% vs. 33%, respectively, *P* = 0.02). A higher number of polymicrobial episodes was observed among patients treated with benzylpenicillin than for treatment with cefuroxime (19% vs. 4%, respectively, *P =* 0.04).

**Table 1:** **Clinical characteristics of*Haemophilus*bacteremia according to definitive antibiotic therapy**^**a**^

		**Definitive antibiotic therapy**			
**Median (interquartile range) or percentage**		**Benzylpenicillin**	**Cefuroxime**	**Aminopenicillins**	**Broadspectrum antibiotics**
**n = 26**	**n = 50**	**n = 12**	**n = 16**
Combination therapy with gentamicin or ciprofloxacin		34.6% (9/26)	22.0% (11/50)	25.0% (3/12)	43.8% (7/16)
Gender (female)		46.2% (12/26)	40.0% (20/50)	58.3% (7/12)	37.5% (6/16)
Age		73 (58–84) (n = 26)	69 (52–76) (n = 50)	62 (26–82) (n = 12)	68 (56–79) (n = 16)
Charlson index					
	Low (0)	57.7% (15/26)	36.0% (18/50)	58.3% (7/12)	25.0% (4/16)
	Medium (1–2)	30.8% (8/26)	48.0% (24/50)	25.0% (3/12)	56.2% (9/16)
	High (>2)	11.5% (3/26)	16.0% (8/50)	16.7% (2/12)	18.8% (3/16)
Smoking		45.8% (11/24)	49.0% (24/49)	45.5% (5/11)	43.8% (7/16)
Alcohol abuse		26.1% (6/23)	21.7% (10/46)	9.1% (1/11)	25.0% (4/16)
Immunosuppression		3.8% (1/26) ^d f^	30.0% (15/50)	16.7% (2/12)	31.2% (5/16)
*Haemophilus species*					
	Non-typeable	69.2% (18/26)	76.0% (38/50)	75.0% (9/12)	62.5% (10/16)
	Typeable	15.4% (4/26)	14.0% (7/50)	16.7% (2/12)	25.0% (4/16)
	Other types	15.4% (4/26)	14.0% (7/50)	8.3% (1/12)	12.5% (2/16)
Polymicrobial bacteremia		19.2% (5/26) ^d^	4.0% (2/50)	0% (0/12)	6.2% (1/16)
Acquisition of bacteremia					
	Community-acquired	65.4% (17/26)	56.0% (28/50) ^g^	91.7% (11/12) ^i^	31.2% (5/16)
	Healthcare-related	23.1% (6/26)	34.0% (17/50)	8.3% (1/12)	43.8% (7/16)
	Hospital-acquired	11.5% (3/26)	10.0% (5/50)	0% (0/12)	25.0% (4/16)
Focus of infection					
	Lung	38.5% (10/26)	52.0% (26/50)	58.3% (7/12)	43.8% (7/16)
	Upper respiratory tract	3.8% (1/26)	6.0% (3/50)	16.7% (2/12)	0.0% (0/16)
	Meningitis	0% (0/26) ^f^	0% (0/50) ^h^	0% (0/12)	25.0% (4/16)
	Endocarditis	3.8% (1/26)	2.0% (1/50)	0% (0/12)	6.2% (1/16)
	Hepato-billiary	11.5% (3/26)	8.0% (4/50)	0% (0/12)	6.2% (1/16)
	Miscellaneous	11.5% (3/26)	12.0% (6/50)	16.7% (2/12)	0.0% (0/16)
	Unknown	30.8% (8/26)	20.0% (10/50)	8.3% (1/12)	18.8% (3/16)
Hospital specialty					
	Medical department	69.2% (18/26)	70.0% (35/50)	41.7% (5/12)	75.0% (12/16)
	Surgical department	26.9% (7/26)	16.0% (8/50)	8.3% (1/12)	12.5% (2/16)
	Intensive care unit	0.0% (0/26)	4.0% (2/50)	16.7% (2/12)	12.5% (2/16)
	Other departments ^b^	3.8% (1/26)	10.0% (5/50)	33.3% (4/12)	0.0% (0/16)
Altered mental state		26.9% (7/26)	24.0% (12/50)	25.0% (3/12)	53.3% (8/15)
Temp. <38 °C		30.8% (8/26)	26.0% (13/50)	9.1% (1/11)	14.3% (2/14)
Mean blood pressure (mmHg)		90 (82–102) (n = 25)	91 (84–100) (n = 48)	92 (71–109) (n = 10)	94 (73–103) (n = 15)
Heart rate		100 (79–108) (n = 25)	95 (80–108) (n = 47)	105 (89–136) (n = 10)	94 (84–106) (n =15)
B-hgb (mmol/L)		6.8 (6.4-8.3) (n = 23)	7.3 (6.3-8.3) (n = 43)	7.9 (6.9-8.7) (n = 9)	7.7 (6.0-9.0) (n = 14)
B-WBC (10^9^ cells/L)		13.3 (10.5-16.6) (n = 23)	15.5 (9.7-20.5) (n = 49)	10.6 (7.5-17.7) (n = 10)	8.2 (2.1-18.8) (n = 15)
P-creatinine (μmol/L)		79 (58–137) (n =19)	90 (62–133) (n = 43)	69 (56–99) (n = 9)	105 (71–240) (n = 14)
Abnormal liver parameters ^c^		72.2% (13/18)	54.1% (20/37)	40.0% (2/5)	40.0% (4/10)
P-CRP (mg/L)		145 (45–214) (n = 24) ^d^	184 (105–262) (n = 48)	186 (55–241) (n = 10)	173 (119–271) (n = 14)
Transfer to ICU		11.5% (3/26)	12.2% (6/49) ^h^	25.0% (3/12)	37.5% (6/16)
Septic shock		11.5% (3/26)	0% (0/48) ^h^	8.3% (1/12)	26.7% (4/15)
Mechanical ventilation		7.7% (2/26) ^f^	8.2% (4/49) ^h^	8.3% (1/12)	37.5% (6/16)
Hemodialysis		3.8% (1/26)	2.1% (1/48) ^h^	0% (0/12)	18.8% (3/16)
Death		38.5% (10/26) ^d e^	12.0% (6/50) ^h^	0% (0/12) ^i^	37.5% (6/16)

a) 1 pt with inoperable mouth cancer died without initiation of antibiotic therapy.

b) Paediatric, gynaecological and ear nose and throat departments.

c) Liver parameters were considered abnormal, if P-ALAT > 45 U/L, P-ASAT > 35 U/L, P-Albumin < 550 μM, P-Amylase > 120 U/L, P-Bilirubin > 22 μM or P-alkaline phosphatase > 105 U/L.

Significant difference (Fisher exact test or Mann Whitney test, P < 0.05): benzylpenicillin vs. cefuroxime (d), benzylpenicillin vs. /aminopenicillins (e), benzylpenicillin vs. broadspectrum antibiotics (f), cefuroxime vs. aminopenicillins (g) cefuroxime vs. broadspectrum antibiotics (h). aminopenicillins vs. broadspectrum antibiotics (i).

### Mortality

The overall 30-day CFR was 22% (Non-typeable *H. influenzae* (25.0%), typeable *H. influenzae* (12%), other *Haemophilus* species (17%), see Table 2). Patients with hospital-acquired bacteremia were more likely to die than patients with community-acquired or healthcare-related bacteremia (54% vs. 26% and 19%, respectively, *P <* 0.01). Polymicrobial bacteremic episodes had a significantly higher CFR than monomicrobial episodes (38% vs. 19%, respectively, *P* = 0.01). Alcohol abuse (45% vs. 17%, *P =* 0.01), altered mental state (63% vs. 19%, *P* < 0.01), body temperature < 38 °C (41% vs. 20%, P < 0.05), admittance at intensive care unit (17% vs. 2%, *P* = 0.02), development of septic shock (23% vs. 4%, *P =* 0.01), and need for mechanical ventilation (26% vs. 9%, *P* = 0.04) were all observed more frequently among non-survivors than among survivors, respectively.

**Table 2:** **Clinical characteristics of*Haemophilus*bacteremia according to 30-day CFR**

	**Median (interquartile range) or percentage**	**Non-survivors**	**Survivors**	**OR**	**P-value**
		**n = 23**	**n = 82**		
Gender (female)		47.8% (11/23)	41.5% (34/82)	1.29 (0.51-3.28)	0.64
Age		69 (60–83)	69 (50–77)	1.03 (1.00-1.05)	0.07
	0-64 years	34.8% (8/23)	46.3% (38/82)	1 (reference)	
	65-80 years	26.1% (6/23)	35.4% (29/82)	0.98 (0.31-3.15)	0.98
	>80 years	39.1% (9/23)	18.3% (15/82)	2.85 (0.93-8.77)	0.07
*Haemophilus* species					
	Nontypeable	82.6% (19/23)	69.5% (57/82)	1 (reference)	
	Typeable	8.7% (2/23)	18.3% (15/82)	0.40 (0.08-1.91)	0.25
	Others	8.7% (2/23)	12.2% (10/82)	0.60 (0.12-2.99)	0.53
Polymicrobial bacteremia		21.7% (5/23)	3.7% (3/82)	7.32 (1.60-33.5)	**0.01**
Charlson index					
	Low (0)	30.4% (7/23)	45.1% (37/82)	1 (reference)	
	Medium (1–2)	52.2% (12/23)	39.0% (32/82)	1.98 (0.70-5.64)	0.20
	High (>2)	17.4% (4/23)	15.9% (13/82)	1.63 (0.41-6.47)	0.49
Smoking		50.0% (11/22)	46.8% (37/79)	1.14 (0.44-2.92)	0.81
Alcohol abuse		45.0% (9/20)	16.9% (13/77)	4.03 (1.40-11.67)	**0.01**
Immunosuppression		13.6% (3/22)	24.1% (20/83)	0.50 (0.13-1.86)	0.39
Focus of infection					
	Lung	52.2% (12/23)	46.3% (38/82)	1.29 (0.50-3.19)	0.64
	Upper respiratory tract	0.0% (0/23)	7.3% (6/82)	0.25 (0.01-4.62)	0.34
	Meningitis	8.7% (2/23)	2.4% (2/82)	3.81 (0.51-28.67)	0.21
	Endocarditis	0.0% (0/23)	3.7% (3/82)	0.48 (0.02-9.70)	>0.99
	Hepato-billiary	4.3% (1/23)	9.8% (8/82)	0.42 (0.05-3.55)	0.68
	Miscellaneous	4.3% (1/23)	12.2% (10/82)	0.33 (0.04-2.70)	0.45
	Unknown	30.4% (7/23)	19.5% (16/82)	1.81 (0.64-5.12)	0.27
Acquisition of bacteremia					
	Community-acquired	43.5% (10/23)	62.2% (51/82)	1 (reference)	
	Healthcare-related	26.1% (6/23)	30.5% (25/82)	1.22 (0.40-3.75)	0.72
	Hospital-acquired	30.4% (7/23)	7.3% (6/82)	5.95 (1.65-21.48)	**<0.01**
Hospital specialty					
	Medical	56.5% (13/23)	69.5% (57/82)	1 (reference)	
	Surgical	21.7% (5/23)	15.9% (13/82)	1.69 (0.51-5.57)	0.39
	Intensive care unit	17.4% (4/23)	2.4% (2/82)	8.78 (1.45-53.1)	**0.02**
	Others^a^	4.3% (1/23)	12.2% (10/82)	0.44 (0.05-3.73)	0.45
Altered mental state		65.2% (15/23)	18.5% (15/81)	8.25 (2.96-23.00)	**<0.01**
Temp. <38		40.9% (9/22)	20.0% (16/80)	2.77 (1.01-7.61)	**<0.05**
Mean blood pressure (mmHg)		97 (86–113) (n = 22)	90 (81–100) (n = 76)	1.03 (1.00-1.06)	0.15
Heart rate		100 (84–112) (n = 22)	95 (80–108) (n = 76)	1.01 (0.99-1.03)	0.46
B-hgb (mmol/L)		7.3 (6.3-9.0) (n = 21)	7.3 (6.5-8.4) (n = 69)	1.08 (0.75-1.55)	0.7
B-WBC (10^9^ cells/L)		13.3 (11.6-17.6) (n = 23)	14.2 (8.3-20) (n = 75)	0.99 (0.93-1.05)	0.61
P-creatinine (μmol/L)		91 (61–141) (n = 21)	82 (61–120) (n = 64)	1.00 (0.99-1.01)	0.97
Abnormal liver parameters^b^		58.8% (10/17)	54.7% (29/53)	1.18 (0.39-3.58)	>0.99
P-CRP (mg/L)		175 (96–270) (n = 21)	175 (97–257) (n = 76)	1.00 (1.00-1.01)	0.97
Transfer to ICU		30.4% (7/23)	13.6% (11/81)	2.78 (0.93-8.30)	0.11
Shock		22.7% (5/22)	3.75% (3/80)	7.55 (1.64-34.68)	**0.01**
Mechanical ventilation		26.1% (6/23)	8.6% (7/81)	3.73 (1.11-12.53)	**0.04**
Hemodialysis		4.5% (1/22)	4.9% (4/81)	0.92 (0.10-8.64)	>0.99
Inadequate empiric antibiotic therapy		8.7% (2/23)	12.2% (10/82)	0.69 (0.14-3.38)	>0.99
Definitive antibiotic therapy					
Benzylpenicillin		45.5% (10/22)	19.5% (16/82)	1 (reference)	
Aminopenicillin		0% (0/22)	14.6% (12/82)	Not determined^c^	**<0.02**^**d**^
Cefuroxime		27.2% (6/22)	53.7% (44/82)	0.22 (0.07-0.70)	**0.01**
Broadspectrum antibiotics		27.2% (6/22)	12.2% (10/82)	0.96 (0.27-3.47)	0.95

a) Paediatric, gynaecological and ear nose and throat departments.

b) Liver parameters were considered abnormal, if P-ALAT > 45 U/L, P-ASAT > 35 U/L, P-Albumin < 550 μM, P-Amylase > 120 U/L, P-Bilirubin > 22 μM or P-alkaline phosphatase > 105 U/L.

c) OR could not be determined, because CFR = 0 for aminopenicillins.

d) P-value was determined by Fisher Exact test for benzylpenicillin vs. aminopenicillins.

#### Mortality and empiric antibiotic therapy

32% (7/22) of the patients initially given benzylpenicillin as monotherapy died, and when combined with gentamicin, the 30-day CFR was 27% (4/15). None of the patients receiving aminopenicillins died (0/7), whereas 19% (6/31) of the patients receiving cefuroxime as monotherapy died, and when combined with another antibiotic, none of the patients died (0/10). The 30-day CFR for empiric therapy with broad-spectrum antibiotics was 42% (piperacillin-tazobactam: 40% (2/5), 3. generation cephalosporins: 50% (2/4), meropenem: 100% (1/1), ciprofloxacin: 0% (0/1) and gentamicin: 0% (0/1)). Thus, empiric antibiotic treatment with cefuroxime/aminopenicillins resulted in a lower CFR than therapy with benzylpenicillin (13% (6/48) vs. 30% (11/37), respectively, *P =* 0.06) or with broadspectrum antibiotics (13% vs. 38% (5/13), respectively, *P* < 0.05). 22% (2/9) of patients not receiving adequate empiric antibiotic therapy died (1 treated with benzylpenicillin, who was infected with an ampicillin-resistant non-typeable *H. influenzae*, and 1 did not receive antibiotics), whereas 21 out of 96 patients (22%) receiving adequate antibiotic therapy died (*P* > 0.05). If monotherapy benzylpenicillin, in contrast to our initial definition, was considered as an inadequate empiric therapy, the CFR was 26% (8/30) for inadequate therapy and 20% (15/75) for adequate therapy (*P* > 0.05). The CFR was not significantly associated to whether the patients received empiric treatment as monotherapy or combination therapy (23% (15/65) vs. 19% (7/36), respectively, *P* > 0.05).

#### Mortality and definitive antibiotic therapy

**Figure 1:**
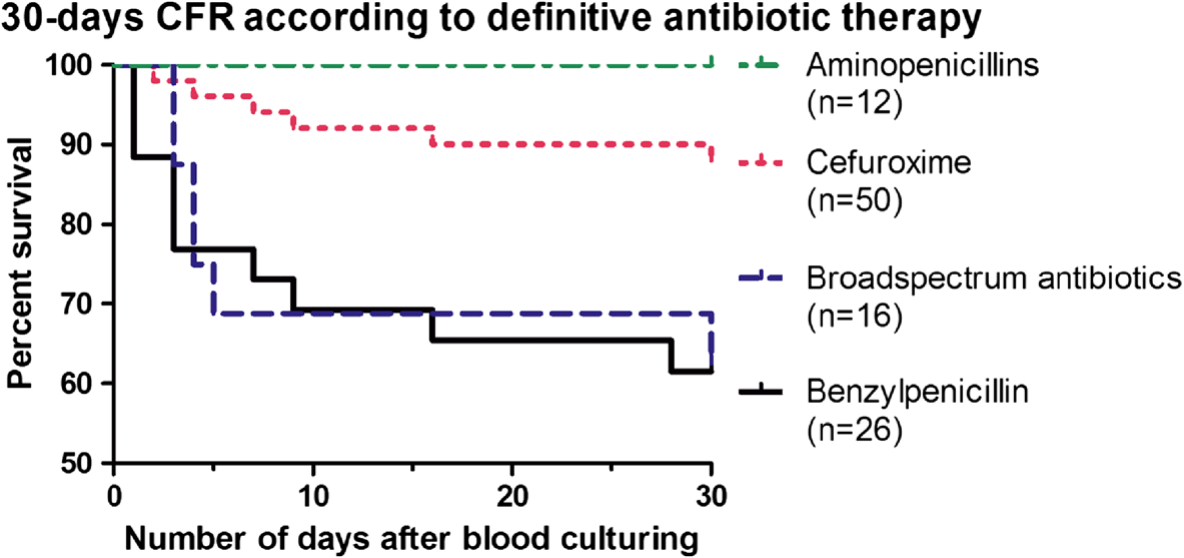
**Kaplan Meyer Survival curves for Haemophilus bacteremia according to definitive antibiotic therapy.** Treatment with cefuroxime or aminopenicillins resulted in a significant lower 30-day CFR than treatment with benzylpenicillin or broadspectrum antibiotics (Log rank test, *P <* 0.02). When compared to benzylpenicillin, treatment with cefuroxime caused a significantly lower 30-day CFR in crude analysis (OR: 0.22 (0.07-0.70, *P =* 0.01), and in adjusted analyses including variables presented in Table 2 (Bivariate logistic regression, *P* < 0.05).

When therapy with cefuroxime or aminopenicillins was chosen after result of susceptibility testing, the CFR was significantly lower than for therapy with benzylpenicillin (12% (6/50) or 0% (0/12) vs. 39% (10/26), respectively or to therapy with broadspectrum antibiotics 38% (6/16) (Log rank test, *P <* 0.02, see Figure 1). The CFR was not significantly associated with the use of mono- or combination therapy (19% (14/74) vs. 27% (8/30), respectively, *P =* 0.4). When adjusting for other identified risk factors in bivariate Logistic regression analysis (see Table 2), treatment with cefuroxime was still associated with a significantly lower CFR than for benzylpenicillin: OR: 0.21 (0.06-0.69), *P =* 0.01 (hospital-acquired bacteremia), OR: 0.27 (0.08-0.91), *P* < 0.04 (polymicrobial episodes), OR: 0.16 (0.04-0.59), *P =* 0.006 (admittance at intensive care unit), OR: 0.22 (0.06-0.82), *P =* 0.02 (alcohol abuse), OR: 0.15 (0.04-0.60), *P =* 0.008 (altered mental state), OR: 0.22 (0.07-0.71), *P =* 0.01 (temperature < 38 °C), OR: 0.23 (0.07-0.79), *P =* 0.02 (septic shock), OR: 0.21 (0.06-0.69), *P =* 0.01 (mechanical ventilation). Also when adjusted for the other co-factors shown in Table 2 (e.g. combination therapy with gentamicin/ciprofloxacin, age, gender, Charlson index, smoking, immunosuppression, focus of infection, type of *Haemophilus* species) in bivariate analysis, treatment with benzylpenicillin was still associated with a significantly higher CFR than therapy with cefuroxime/aminopenicillins (data not shown, *P <* 0.05). Beside this, if all analyses were performed for *H. influenzae* only or if the patient, who were treated with benzylpenicillin and did not receive adequate definitive antibiotic therapy, was excluded from the analyses, treatment with benzylpenicillin was still associated with a significantly higher CFR than treatment with cefuroxime/aminopenicillins (data not shown, *P* < 0.05).

## Discussion

In the present study we found that the CFR was significantly higher when treatment with benzylpenicillin was continued as definitive therapy after the result of susceptibility testing, when compared to treatment with cefuroxime/aminopenicillins. When adjusting for other identified risk factors or other co-factors in bivariate Logistic regression analysis, a significantly higher risk of dying was still observed after therapy with benzylpenicillin as compared to cefuroxime. Moreover, empiric treatment with cefuroxime/aminopenicillins resulted in lower CFR compared to therapy with benzylpenicillin, although the difference did not reach statistical significance (*P =* 0.06). To our knowledge this is the first study investigating the treatment efficacy of benzylpenicillin in *Haemophilus* bacteremia. Given the lack of research in this area, EUCAST has considered that there are insufficient data for *H. influenzae* to set clinical breakpoints for benzylpenicillin, whereas other guidelines until recently have suggested 1/1 mg/L (SRGA) or 1/4 mg/L (The Norwegian Working Group on Antibiotics (http://www.unn.no/afa) as the breakpoints (S≤/R>) for benzylpenicillin [5]. Interestingly, the MIC values and distribution for *H. influenzae* and *H. parainfluenzae* for benzylpenicillin, aminopenicillin and cefuroxime are comparable, and time-kill experiments have demonstrated benzylpenicillin to be as active as amoxicillin against various *Haemophilus* species [4]. In contrast, the pharmacokinetic profile is more favorable with aminopenicillins/cefuroxime than with benzylpenicillin due to the former’s lower protein binding (17-40% vs. ~45-65%, respectively) and longer serum elimination half-life (*T*_½_: 1.1-1.4 vs. 0.5-0.75 hrs, respectively) (http://www.eucast.org, [6,7]). Indeed, EUCAST has calculated significantly lower target attainment rates (i.e. time above the minimal inhibitory concentration-target of 30-40% of the dosing interval above MICs for susceptible *H. influenzae* population) for benzylpenicillin (1.2 g qid) than for amoxicillin (1 g qid)/cefuroxime (1.5 g tid) as determined by Monte Carlo simulations (e.g. 46-76% vs. 100%, respectively for MIC: 1–2 mg/L, T > MIC: 40%). Most likely such less favorable pharmacodynamic properties with benzylpenicillin therapy could potentially explain the significantly higher CFR of *Haemophilus* bacteremia with the dosing used in the present study (benzylpenicillin: 1.2 g qid). Thus, our results suggest that treatment of adults with benzylpenicillin in doses of 1.2 qid should not be considered as therapy for *Haemophilus* bacteremia, but whether higher doses of benzylpenicillin (e.g. 2.4 g × 6 resulting in target attainment rates of 95-98% for MIC: 1–2 mg/L, T > MIC: 40%) could be an effective treatment still remains to be evaluated.

Our study has some important limitations. Our study was not a randomized study comparing the treatment efficacy of benzylpenicillin and cefuroxime/aminopenicillins for *Haemophilus* bacteremia, and the retrospective study design could potentially have influenced the strength of the statistical calculations due to missing values. Our data collection, however, was almost complete regarding the clinical characteristics and antibiotic therapy. Multivariable analysis for significant risk factors for progression to death could not be performed including all the risk factors identified in the univariate analysis, because there was a relatively low number of outcomes compared to number of variables included in the analysis [8]. The definitive antibiotic therapy was therefore only adjusted for single individual risk factors in separate bivariate analyses.

Whereas the epidemiology of *Haemophilus* infections has been studied extensively since the introduction of the *H. influenzae* type b vaccine in childhood vaccination programs [9-11] demonstrating a virtual elimination of invasive *H. influenzae* type b infections [12-16], less is known about the clinical presentation of *Haemophilus* infections in the post-vaccination era. In the present study, we found that the clinical presentation (e.g. focus of infection) of *Haemophilus* bacteremia still followed observations from the pre-vaccination period and was closely related to the *Haemophilus species*. Almost all bacteremic episodes with a lung focus were due to non-typeable *H. influenzae*; meningitis and epiglottis were primarily observed among episodes due to typeable *H. influenzae*, whereas endocarditis was solely due to *H. parainfluenzae*. Non-typeable *H. influenzae* accounted for most of the bacteremic episodes (3 out of every 4 episodes), and in accordance with previous studies, patients infected with non-typeable *H. influenzae* had an higher age, were more often immunosuppressed, and had a high CFR [17-20]. Almost all healthcare-related bacteremic episodes were due to non-typeable *H. influenzae*, whereas patients with typeable *H. influenzae* bacteremia had the youngest median age – a relationship which has also been demonstrated previously [20]. These patients were more frequently transferred to an intensive care unit and received mechanical ventilation, but had a lower CFR than the other patient groups. This was most likely due to a high number of patients with epiglottitis, who all required assisted ventilation, but all survived the bacteremic episode.

## Conclusion

Our results suggest that, after susceptibility testing, cefuroxime/aminopenicillins is preferable to benzylpenicillins as definitive therapy for *H. influenzae* bacteremia.

## Methods

### Identification of bacteremic episodes

All episodes of positive blood cultures with *Haemophilus species* identified at the Department of Clinical Microbiology during the time period 2000–2009 were included in the study. The Department of Clinical Microbiology provides diagnostic service for all hospitals (three public funded university hospitals with approximately 1600 beds) and primary care in the former Copenhagen County (approximately 620,000 inhabitants, 526 km^2^).

Blood culturing was performed at the attending physician’s decision comprising 1 venipuncture per episode (40 ml of blood distributed in two aerobic and two anaerobic bottles for adults and 8 ml in pediatric bottles for small children; BACTEC®, Becton-Dickinson, Sparks, MD, USA). In case of a positive blood culture, a physician of the Department of Clinical Microbiology notified by telephone the treating physician of the patient concerned, giving the results of the Gram staining and the motility examination, and also supplying recommendations about antibiotic therapy, diagnostic and therapeutic procedures etc. Another contact with the treating physician was made the following day to provide the results of susceptibility testing and bacterial identification. The positive blood culture was sub-cultured, and the identification of the various *Haemophilus* strains was done using standard methods [21]; subsequent serotyping was performed using serotype-specific anti-sera and/or was tested at the National Reference Laboratory at Statens Serum Institut. Susceptibility testing was performed by disc diffusion methods (Oxoid, Hanks, UK), according to zone diameter break-points set out by the Swedish Reference Group for Antibiotics (SRGA: http://www.srga.org). β-lactamase production was determined by use of Cefinase™ paper discs (Becton Dickinson, NJ, USA) to identify resistance to benzylpenicillin and aminopenicillins. Cefuroxime-resistance was confirmed by subsequent MIC determination (E-test®, bioMérieux, Marcy l’Etoile, France).

### Data collection

Clinical and laboratory information were collected retrospectively from medical records. Hospital admission and discharge records including date of death were confirmed in the National Hospital Registration Database. The study was approved by the Danish Data Protection Agency (Record 2008-41-2688) and followed the guidelines of the local scientific committee.

### Definitions

The bacteremic episode was defined as community-acquired or hospital-acquired according to CDC criteria [22]. In addition, a healthcare-related group was defined for patients who had been hospitalized within 30 days prior to the bacteremic episode, or who regularly visited the hospital (e.g. for chemotherapy or hemodialysis) [23]. Polymicrobial episodes were defined as isolation of a separate significant agent from blood cultures within two days of the *Haemophilus* bacteremic episode. Hospital departments were divided into medical -, surgical -, intensive care unit (ICU) or other departments (e.g. pediatric, gynaecological and ear-nose-throat departments). The focus of the infection was registered on the basis of clinical, radiological and microbiological findings.

Comorbidity at time of hospitalization was assessed for each individual patient using the Charlson comobidity index scores: 0 points = low, 1–2 points = medium and >2 = high [24]. Patients were defined as being immunosuppressed if they had cancer or had received immunosuppressive therapy. Alcohol abuse was defined as an alcohol intake of more than 14 units per week for women and more than 21 units per week for men. Patients were considered to have either never smoked or smoked at least once. Shock was registered if a patient had clinical evidence of shock (e.g. hypotension and tachycardia) and/or received vasoactive drugs. The case fatality rate (CFR) was defined as the all cause mortality rate within 30 days after the positive blood culture was taken.

Empiric antibiotic therapy was considered adequate if the patient received intravenous treatment with an antibiotic for which the bacterial isolate was fully susceptible, except for ciprofloxacin, where oral therapy was also considered adequate. According to the local guidelines of the Department of Clinical Microbiology, therapy with benzylpenicillin was considered adequate for isolates susceptible for ampicillin. Definitive antibiotic therapy was defined as the antibiotic therapy administered after the results of susceptibility testing were available. If a patient died before susceptibility data were available, the empiric antibiotic therapy given at time of death was also considered as definitive antibiotic therapy.

### Statistics

Statistics were performed using the Statistical Package for Social Sciences (version 16.0; SPSS, IBM). Data are represented as medians and interquartile ranges. Comparison between groups was performed using the Mann Whitney test for continuous data and the Fisher Exact test for categorical data. Kaplan-Meyer survival curves and the Log-rank test were performed according to definitive antibiotic therapy (benzylpenicillin, cefuroxime, aminopenicillins and broadspectrum antibiotics). Logistic regression analysis was employed for calculating the relative risk of dying within 30 days after blood culturing. Due to the relatively low number of fatal outcomes compared to the number of variables studied, multivariable analyses were only performed for evaluating the association between definitive antibiotic therapy and mortality by adjusting for confounding variables in bivariate analyses [8]. *P-*value <0.05 was considered as statistically significant.

## Competing interests

The authors declare that they have no conflict of interest.

## Author’ contributions

CØ designed the study. ST collected data from medical records. CØ + ST performed data analysis, drafted and approved the final manuscript.

## Pre-publication history

The pre-publication history for this paper can be accessed here:

http://www.biomedcentral.com/1471-2334/12/153/prepub

## Supplementary Material

Additional file 1 **Table S1.**Clinical characteristics of the bacteremic episode according to *Haemophilus* grouping. Click here for file
