# Supplementary material for: Treatment of Haemophilus bacteremia with benzylpenicillin is associated with increased (30-day) mortality
Source: BMC Infect Dis. 2012 Jul 9;12:153. doi: 10.1186/1471-2334-12-153 (PMC3407763; doi:10.1186/1471-2334-12-153)
Supplement: Additional file 1 — Table S1.Clinical characteristics of the bacteremic episode according to Haemophilus grouping. [file 1471-2334-12-153-S1.docx]

**Additional file 1: Table S1. Clinical characteristics of the bacteremic episode according to *Haemophilus* grouping**

|  | Median (interquartile range) | | All cases | Nontypeable | Typeable | Others |
| --- | --- | --- | --- | --- | --- | --- |
|  | or percentage | | n = 105 | n = 76 | n = 17 | n = 12 |
| Gender (female) | | | 42.9% (45/105) | 44.7% (34/76) | 41.2% (7/17) | 33.3% (4/12) |
| Age | |  | 69 (54-79) | 73 (56-82) ^c^ | 61 (46-74) | 65 (42-69) |
| Charlson index | | |  |  |  |  |
|  | | Low (0) | 41.9% (44/105) | 36.8% (28/76) | 64.7% (11/17) | 41.7% (5/12) |
|  | | Medium (1-2) | 41.9% (44/105) | 44.7% (34/76) | 29.4% (5/17) | 41.7% (5/12) |
|  | | High (>2) | 16.2% (17/105) | 18.4% (14/76) | 5.9% (1/17) | 16.7% (2/12) |
| Smoking | | | 47.5% (48/101) | 47.9% (35/73) | 37.5% (6/16) | 58.3% (7/12) |
| Alcohol abuse | | | 22.7% (22/97) | 23.9% (17/71) | 13.3% (2/15) | 27.3% (3/11) |
| Immunosuppression | | | 22.0% (23/105) | 27.6% (21/76) | 5.9% (1/17) | 8.3% (1/12) |
| Polymicrobial bacteremia | | | 7.6% (8/105) | 10.5% (8/76) | 0% (0/17) | 0% (0/12) |
| Acquisition | | |  |  |  |  |
|  | | Community-acquired | 58.1% (61/105) | 51.3% (39/76) ^c^ | 82.4% (14/17) | 66.7% (8/12) |
|  | | Healthcare-related | 29.5% (31/105) | 35.5% (27/76) | 17.6% (3/17) | 8.3% (1/12) |
|  | | Hospital-acquired | 12.4% (13/105) | 13.2% (10/76) | 0.0% (0/17) | 25.0% (3/12) |
| Focus | | |  |  |  |  |
|  | | Lung | 47.6% (50/105) | 61.8% (47/76) ^c^ | 5.9% (1/17) | 16.7% (2/12) |
|  | | Upper respiratory tract | 5.7% (6/105) | 1.3% (1/76) | 29.4% (5/17) | 0.0% (0/12) |
|  | | Meningitis | 3.8% (4/105) | 1.3% (1/76) | 17.6% (3/17) | 0.0% (0/12) |
|  | | Endocarditis | 2.9% (3/105) | 0.0% (0/76) ^d^ | 0.0% (0/17) | 25.0% (3/12) |
|  | | Hepato-billiary | 8.6% (9/105) | 9.2% (7/76) | 0.0% (0/17) | 16.7% (2/12) |
|  | | Miscellaneous | 10.5% (11/105) | 5.3% (4/76) | 23.5% (4/17) | 25.0% (3/12) |
|  | | Unknown | 21.9% (23/105) | 21.1% (16/76) | 23.5% (4/17) | 25.0% (3/12) |
| Hospital specialty | | |  |  |  |  |
|  | | Medical | 66.7% (70/105) | 71.1% (54/76) | 47.1% (8/17) | 66.7% (8/12) |
|  | | Surgical | 17.1% (18/105) | 17.1% (13/76) | 11.8% (2/17) | 25.0% (3/12) |
|  | | Intensive care unit | 5.7% (6/105) | 5.3% (4/76) | 11.8% (2/17) | 0.0% (0/12) |
|  | | Others^a^ | 10.5% (11/105) | 6.6% (5/76) ^c^ | 29.4% (5/17) | 8.3% (1/12) |
| Altered mental state | | | 28.8% (30/104) | 28.9% (22/76) | 25.0% (4/16) | 33.3% (4/12) |
| Temp. <38ºCelsius | | | 24.5% (25/102) | 22.4 % (17/76) | 28.6% (4/14) | 33.3% (4/12) |
| Mean blood pressure (mmHg) | | | 91 (83-102) (n = 98) | 93 (82-103) (n = 73) | 87 (81-98) (n = 14) | 93 (83-97) (n = 11) |
| Heart rate | | | 96 (80-108) (n = 98) | 97 (83-109) (n = 72) | 90 (73-105) (n = 14) | 88 (77-100) (n = 12) |
| B-hgb (mmol/L) | | | 7.3 (6.4-8.4) (n = 90) | 7.3 (6.4-8.4) (n = 64) | 7.2 (6.9-8.4) (n = 17) | 6.1 (5.5-8.4) (n = 9) |
| B-WBC (10^9^ cells/L) | | | 13.8 (9.4-19.1) (n = 98) | 13.7 (9.4-18.8) (n = 71) | 15.2 (8.1-21.1) (n = 17) | 14.0 (8.7-18.5) (n = 10) |
| P-creatinine (µmol/L) | | | 83 (61-135) (n = 85) | 85 (59-136) (n = 60) | 82 (59-120) (n = 17) | 94 (72-156) (n = 8) |
| Abnormal liver parameters^b^ | | | 55.7% (39/70) | 52.0% (26/50) | 57.1% (8/14) | 83.3% (5/6) |
| P-CRP (mg/L) | | | 175 (97-258) (n = 97) | 165 (92-256) (n = 70) | 221 (157-272) (n = 17) | 186 (108-230) (n = 10) |
| Transfer to ICU | | | 17.3% (18/104) | 13.2% (10/76) ^c^ | 47.1% (8/17) ^e^ | 0.0% (0/11) |
| Shock | | | 7.8% (8/102) | 8.0% (6/75) | 6.2% (1/16) | 9.1% (1/11) |
| Mechanical ventilation | | | 12.5% (13/104) | 11.8% (9/76) | 23.5% (4/17) | 0.0% (0/12) |
| Hemodialysis | | | 4.9% (5/103) | 5.3% (4/75) | 5.9% (1/17) | 0.0% (0/11) |
| Death | | | 21.9% (23/105) | 25.0% (19/76) | 11.8% (2/17) | 16.7% (2/12) |

a) Paediatric, gynaecological and ear nose and throat departments.

b) Liver parameters were considered abnormal, if P-ALAT > 45 U/L, P-ASAT > 35 U/L, P-Albumin < 550 µM, P-Amylase > 120 U/L, P-Bilirubin > 22 µM or P-alkaline phosphatase > 105 U/L.

c) P-value < 0.05 non-typeable vs. typeable,

d) P-value < 0.05 non-typeable v. others

e) P-value < 0.05 typeable vs. others
